# Evolution of mobile genetic element composition in an epidemic methicillin-resistant *Staphylococcus aureus*: temporal changes correlated with frequent loss and gain events

**DOI:** 10.1186/s12864-017-4065-z

**Published:** 2017-09-04

**Authors:** Dorota Jamrozy, Francesc Coll, Alison E. Mather, Simon R. Harris, Ewan M. Harrison, Alasdair MacGowan, Andreas Karas, Tony Elston, M. Estée Török, Julian Parkhill, Sharon J. Peacock

**Affiliations:** 1 The Wellcome Trust Sanger Institute, Wellcome Trust Genome Campus, Hinxton, Cambridge, CB10 1SA UK; 2 London School of Hygiene and Tropical Medicine, London, WC1E 7HT UK; 3 Department of Veterinary Medicine, University of Cambridge, Cambridge, CB3 0ES UK; 4 Department of Medicine, University of Cambridge, Addenbrooke’s Hospital, Cambridge, CB2 0QQ UK; 5 British Society for Antimicrobial Chemotherapy, B1 3NJ, Birmingham, UK; 6 Public Health England, Clinical Microbiology and Public Health Laboratory, Cambridge, CB21 5XA UK; 7 Colchester Hospital University NHS Foundation Trust, Colchester, CO4 5JL UK; 8 Cambridge University Hospitals NHS Foundation Trust, Cambridge, CB2 0QQ UK

**Keywords:** *Staphylococcus aureus*, HA-MRSA, EMRSA-15, CC22, Evolution, MGE, Horizontal gene transfer

## Abstract

**Background:**

Horizontal transfer of mobile genetic elements (MGEs) that carry virulence and antimicrobial resistance genes mediates the evolution of methicillin-resistant *Staphylococcus aureus,* and the emergence of new MRSA clones. Most MRSA lineages show an association with specific MGEs and the evolution of MGE composition following clonal expansion has not been widely studied.

**Results:**

We investigated the genomes of 1193 *S. aureus* bloodstream isolates, 1169 of which were MRSA, collected in the UK and the Republic of Ireland between 2001 and 2010. The majority of isolates belonged to clonal complex (CC)22 (*n* = 923), which contained diverse MGEs including elements that were found in other MRSA lineages. Several MGEs showed variable distribution across the CC22 phylogeny, including two antimicrobial resistance plasmids (pWBG751-like and SAP078A-like, carrying erythromycin and heavy metal resistance genes, respectively), a pathogenicity island carrying the enterotoxin C gene and two phage types Sa1*int* and Sa6*int*. Multiple gains and losses of these five MGEs were identified in the CC22 phylogeny using ancestral state reconstruction. Analysis of the temporal distribution of the five MGEs between 2001 and 2010 revealed an unexpected reduction in prevalence of the two plasmids and the pathogenicity island, and an increase in the two phage types. This occurred across the lineage and was not correlated with changes in the relative prevalence of CC22, or of any sub-lineages within in.

**Conclusions:**

Ancestral state reconstruction coupled with temporal trend analysis demonstrated that epidemic MRSA CC22 has an evolving MGE composition, and indicates that this important MRSA lineage has continued to adapt to changing selective pressure since its emergence.

**Electronic supplementary material:**

The online version of this article (doi:10.1186/s12864-017-4065-z) contains supplementary material, which is available to authorized users.

## Background

*Staphylococcus aureus* is a major cause of human disease worldwide. The pathogenesis of *S. aureus* infection has been associated with a range of chromosomally-encoded virulence factors. These include exotoxins that damage host cell membranes such as haemolysins and phenol soluble modulins (PSMs), and immune evasion molecules such as protein A and aureolysin [1, 2]. However, the success of *S. aureus* as a pathogen has also been facilitated by its capacity to rapidly evolve through horizontal gene transfer. Mobile genetic elements (MGEs) mediate the acquisition of novel virulence factors and are associated with some of the most potent *S. aureus* virulence molecules, including Panton-Valentine leukocidin and toxic shock syndrome toxin [3]. Equally important is the role of MGEs in accelerating *S. aureus* adaptation to environmental pressures through transfer of antimicrobial resistance genes. The most clinically significant example is the Staphylococcal Cassette Chromosome *mec* (SCC*mec*) element, which carries the *mecA/C* gene encoding methicillin resistance [4].

The widespread clinical use of β-lactam antibiotics and resulting selective pressure for resistance has been associated with the global emergence and spread of SCC*mec* in *S. aureus*, which has been instrumental in the evolution and success of this pathogen in recent years [5]. Multi-locus sequence typing (MLST) has demonstrated that MRSA isolates belong to a limited number of clonal complexes (CCs) [6]. More recently, whole-genome sequencing (WGS) and reconstruction of phylogenetic relationships between MRSA isolates derived from the same CC has demonstrated that MRSA has become widespread predominantly through a process of clonal expansion [7–9]. Several major MRSA clones have emerged, which involved independent SCC*mec* acquisitions by distinct *S. aureus* lineages such as CC5, CC8, CC22, CC30 and CC45 [10]. However, acquisition of SCC*mec* is not the sole event involved in the emergence of MRSA clones. Other evolutionary changes occur such as the acquisition of additional MGEs that collectively constitute molecular markers of a new MRSA clone [11].

Contemporary MRSA clones include the epidemic MRSA-15 (EMRSA-15), which belongs to CC22. The first reported isolation of EMRSA-15 was in the UK in the early 1990s, and it has since become the dominant hospital-associated MRSA (HA-MRSA) in the country [12, 13]. EMRSA-15 subsequently spread beyond the UK, with rapid expansion across Europe to become the dominant HA-MRSA lineage in Australia and Singapore [14–16]. Whole genome analysis of CC22 isolates did not reveal a single prominent genetic element that could explain the success of EMRSA-15 clone, with a combination of genetic variations observed, of which the most notable were determinants of antimicrobial resistance [9]. Reconstruction of evolutionary events that led to the emergence of EMRSA-15 revealed acquisition of mutations in *gyrA*/*grlA* genes conferring resistance to fluoroquinolones followed by a temporal increase in the average number of resistance markers [9]. Additionally, EMRSA-15 was found to suffer a lower fitness cost due to fluoroquinolone resistance than other MRSA clones [17].

The use of WGS to study bacterial pathogens is a rapidly expanding field and has given unprecedented insights into the evolution and epidemiology of species such as *S. aureus*. WGS has provided access to bacterial pan-genomes, which facilitates the non-targeted analysis of genomic diversity. This is particularly relevant to *S. aureus* as MGEs represent around 20% of its total genome content [18]. MGEs can be rapidly lost and acquired, and WGS can now be used to study the flux of MGEs in bacterial populations to gain better understanding of how their evolutionary trajectory changes with the rise and fall of epidemic clones. Here, using WGS data of 1193 *S. aureus* bloodstream isolates collected in the UK and the Republic of Ireland between 2001 and 2010, we conducted a broad analysis of MGE carriage by the dominant MRSA CC22. We defined MGE diversity within the MRSA CC22 population, and investigated instances of inter-lineage MGE distribution involving CC22 and other co-circulating MRSA lineages. We identified several MGEs that were variably distributed across the CC22 phylogeny due to frequent loss and gain events. Temporal changes in MGE carriage were observed, which included a fall in prevalence of elements associated with antimicrobial resistance and virulence. Gaining novel insights into the evolution of MGE composition within an epidemic MRSA population could provide answers relevant to the control of antimicrobial resistance and more broadly reveal evolutionary strategies employed by the highly successful lineages such as MRSA CC22.

## Results

### Collection overview

The analysis was conducted on 1193 *S. aureus* bloodstream isolates, the majority of which were MRSA (*n* = 1169, 98%; see Additional file 1: Table S1). Isolates were sampled between 2001 and 2010 across the UK (England, Wales, Scotland and Northern Ireland) and the Republic of Ireland, providing a broad temporal and regional overview of the population structure of MRSA derived from invasive disease. The population consisted of 14 clonal complexes, the dominant CC being CC22 (*n* = 923, 77%) followed by CC30 (*n* = 180, 15%). Less common were CC5 (*n* = 30 2.5%), CC8 (*n* = 25, 2.1%) and CC1 (*n* = 12, 1.0%). The remaining nine CCs were represented by less than 1% of all isolates and consisted of CC9, CC15, CC20, CC25, CC45, CC59, CC80, CC97 and CC779. Only CC5, CC22 and CC30 were detected in every sampling year. A temporal trend was observed for the distribution of CC22 and CC30, with an increase in the prevalence of CC22 and a fall in the sampling of CC30 over time (see Additional file 2: Figure S1). This is consistent with previous findings for MRSA in the UK [13, 19].

The phylogenetic relationships between CC22 isolates were reconstructed based on a core genome alignment that contained 22,852 SNP sites. This revealed that the CC22 population was composed predominantly of the EMRSA-15 clone (914/923, 99%; see Additional file 2: Figure S2). The phylogeny of CC30 isolates was reconstructed based on a core genome alignment that contained 6227 SNP sites and, similarly to the findings for CC22, the CC30 lineage was dominated by representatives of an epidemic MRSA clone, EMRSA-16 (176/180, 98%, see Additional file 2: Figure S2).

### Mobile genetic elements in MRSA CC22

Whole-genome assemblies of all MRSA CC22 isolates were evaluated for the presence of MGEs. Sequence fragments were categorised as putative SCC elements, pathogenicity islands, transposons or plasmids based on the sequence, and/or insertion site if chromosomal. The carriage of phages was evaluated based on the presence of phage-associated integrase genes, as described previously [20]. Analysis of entire phage genomes from short reads was not possible due to assembly across multiple contigs resulting from their mosaic structure.

All of the MGEs identified are described in Additional file 3: Table S2. Across all CC22 isolates a total of 37 distinct MGE sequences were identified together with five distinct phage types. Elements that were highly prevalent in CC22 consisted of a chromosomally integrated Tn*552*-like transposon carrying the penicillin resistance *blaZ* gene (*n* = 923, 100%), and methicillin resistance determinant SCC*mec* IVh (*n* = 905, 98%). Also common was a pathogenicity island carrying the enterotoxin C gene *sec* (*n* = 564, 61%). This island (referred to as SaPI*sec*) shared complete sequence identity with a region of the CC22 reference genome strain H-EMRSA15 (GenBank CP007659). Most isolates also revealed carriage of two antimicrobial resistance plasmids: a 2.5 kb pWBG751-like element carrying the erythromycin resistance gene *ermC* (*n* = 665, 72%), and a 32 kb SAP078A-like plasmid carrying a cluster of heavy metal resistance genes composed of *cadA*, *copB*, *mco* and *arsAB* (*n* = 499, 54%). For the purpose of this work the pWBG751-like and SAP078A-like plasmids are referred to as P1-*ermC* and P2-hm (heavy metal), respectively.

The remaining elements were found at a frequency of between 0.1% (single isolate only) to 3.7% (*n* = 34). A further four pathogenicity island elements were detected in CC22 including SaPI2 carrying toxic shock syndrome toxin *tst* [21] and SaPI4 [22], although each was found in no more than 10 CC22 isolates. While the majority of CC22 isolates carried SCC*mec* type IVh, other types (IVa, IVc and V) were also detected. Carriage of arginine catabolic mobile element (ACME) type II was detected in 34 CC22 isolates. The ACME element typically forms a composite island together with SCC*mec* (ACME-SCC*mec*-CI) located within the *orfX* gene [23]. A previous report of ACME type II in CC22 described two variants of ACME-SCC*mec*-CI, where ACME was associated with either SCC*mec* type IVa or type IVh, the latter being the most common [24]. Here, we made a similar observation in that 10 of the ACME positive CC22 isolates carried SCC*mec* IVa and the remaining 24 contained SCC*mec* type IVh. Other composite islands within the *orfX* gene were also observed, including two distinct SCC elements of unknown function as well as SCC*fus*, which contains fusidic acid resistance gene *fusC*. All SCC elements were observed in SCC*mec* IVh positive isolates.

In addition to the broadly distributed P1-*ermC* and P2-hm plasmids, 21 further putative plasmids or plasmid fragments were detected in CC22, most of which carried either a single or multiple antimicrobial resistance determinants. Distinct elements carrying homologous resistance genes were detected. In particular, resistance genes *ileS* (mupirocin resistance gene), *tetK* (tetracycline resistance gene) and *qacC* (multidrug efflux protein gene) were each associated with at least 3 different elements. Other resistance genes found on putative plasmids included *catA* (chloramphenicol resistance gene), *aadD* (kanamycin resistance gene) and *bleO* (bleomycin resistance gene). However, all were very infrequent in CC22 with each found in no more than 6 isolates, which may indicate transient carriage by this population.

Analysis of phage type distribution revealed that the majority of CC22 isolates carried Sa2*int* (*n* = 887, 96%) and Sa3*int* (*n* = 850, 92%) phage types. Based on mapping to a complete reference sequence of Sa2*int* phage present in CC22 strain HO 5096 0412, most Sa2*int*-positive isolates (883/887) carried a closely related variant (mapping sequence identity 95–100%, coverage 92–100%). Of the remaining 4 isolates, two carried Sa2*int* phage containing the Panton-Valentine leukocidin gene *pvl* although neither were the EMRSA-15 clone. All but one Sa3*int*-positive CC22 isolates (849/850) carried phage that shared high sequence identity with the Sa3*int*-carried by the CC22 strain HO 5096 4012 (mapping sequence identity 95–100%, coverage 91–100%). The CC22 population also contained a lower prevalence of phages Sa1*int* (*n* = 172, 19%) and Sa6*int* (*n* = 167, 18%).

### Distribution of MGEs identified in MRSA CC22 across *S. aureus* population

**Fig. 1:**
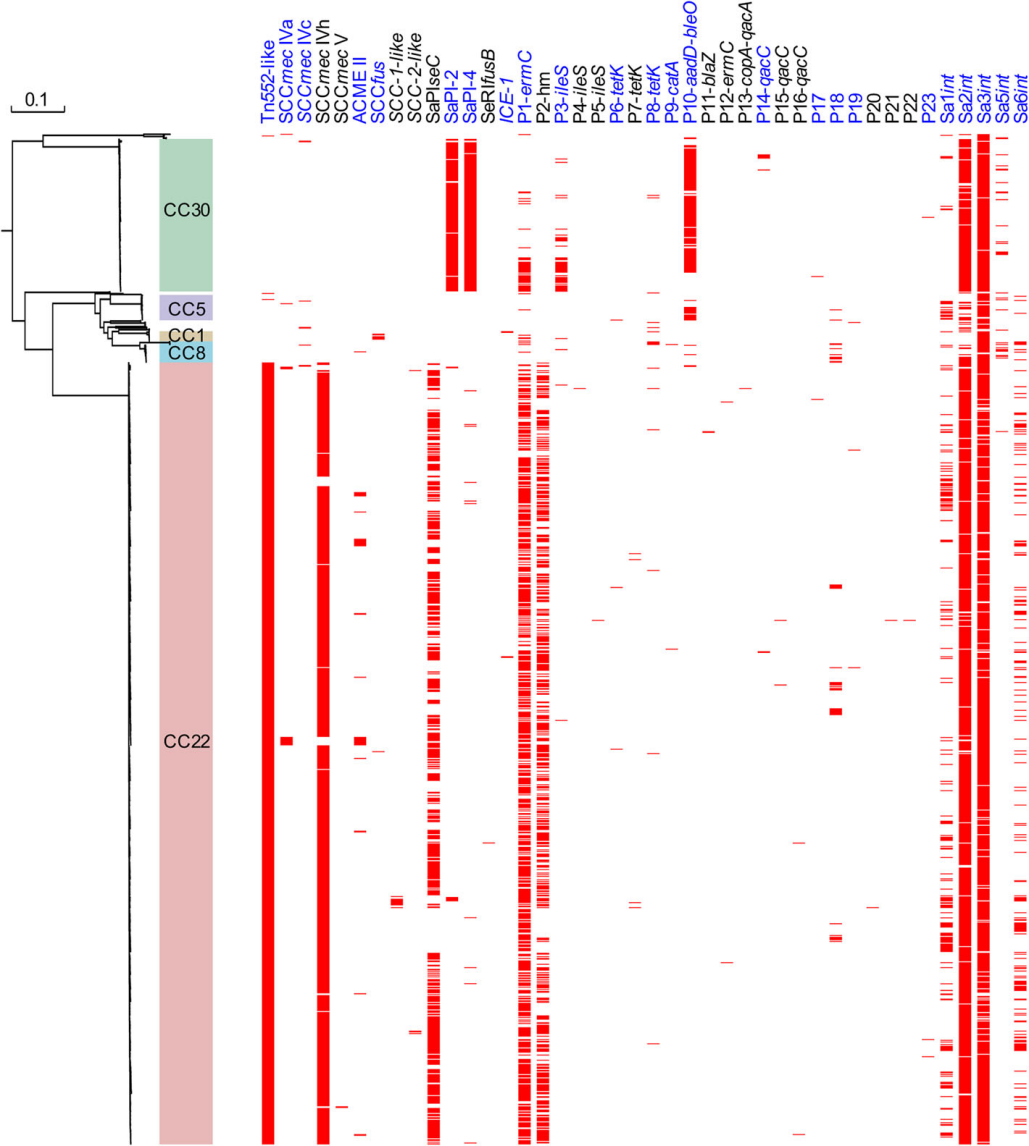
Phylogeny of 1193 *S. aureus* bloodstream isolates and distribution of analysed MGEs. The colour-coded column indicates clades representing the most common lineages: CC1, CC5, CC8, CC22 and CC30. All isolates were screened for carriage of MGEs detected in MRSA CC22. A rooted approximately maximum-likelihood phylogenetic tree was annotated with the distribution of all identified MGEs. Red horizontal lines indicate the presence of MGE. Names of MGEs detected in both CC22 and non-CC22 isolates are highlighted in blue. Putative plasmids were assigned a numeric ID plus the name of antimicrobial resistance gene (if applicable). For chromosomally integrated elements, ID names were assigned based on close homology to a previously described element as identified with Basic Local Alignment Search Tool (BLAST) based on sequence identity cut-off of 99%, or a numeric ID was assigned (name in italics)

We investigated whether MGEs that were detected in MRSA CC22 isolates also occur in representatives of other lineages. The distribution of all identified MGEs is presented in Fig. 1, which shows the phylogeny of all 1193 *S. aureus* isolates based on core genome alignment consisting of 160,857 SNP sites. Amongst the 37 MGEs that were detected in CC22, 19 were also observed in other CCs, which is consistent with inter-lineage dissemination. Specific elements were observed to be common to several CCs. The most frequently shared MGEs among different lineages were small antimicrobial resistance plasmids such as P1-*ermC*, the *tetK*-positive plasmid P8, and the *aadD-bleO*-positive plasmid P10. Despite a low overall prevalence, SCC*mec* type IVc was detected in four non-CC22 lineages. Interestingly, we also observed instances where an element identified in CC22 was notably more prevalent in a non-CC22 lineage. This included pathogenicity islands SaPI-2 and SaPI-4 that were highly prevalent in CC30 (96% and 98%, respectively), SCC*fus* observed in over half of CC1 isolates, and the *addD*-*bleO*-positive plasmid P10, which was present in the majority of CC30 and over half of CC5 isolates. The sporadic acquisition of diverse MGEs might represent a unique feature of CC22. Nevertheless, our data reveals that a number of chromosomally integrated as well as extra-chromosomal elements that have a particular association with a specific lineage can be also infrequently observed in another CC.

### MGE distribution in the context of MRSA CC22 phylogeny

**Fig. 2:**
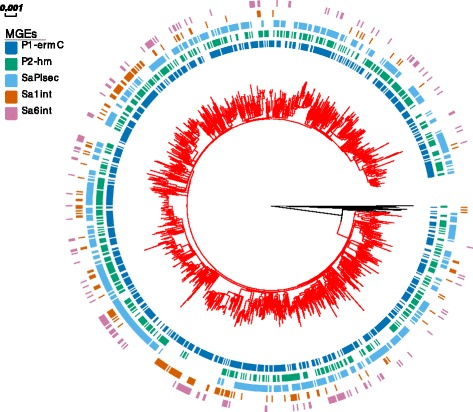
Phylogeny of MRSA CC22 isolates and MGE carriage. A rooted maximum-likelihood phylogenetic tree was annotated with the distribution of selected MGEs: P1-*ermC*, P2-hm, SaPI*sec*, Sa1*int* and Sa6*int*. Branches of clade representing the EMRSA-15 population are shown in red

The variable frequency of certain MGEs in MRSA CC22 provided an opportunity to investigate the evolutionary dynamics of MGE acquisition and maintenance in this population over a 10-year period. For this we focused on the MGEs that were frequent but not uniformly present in CC22, namely plasmids P1-*ermC* and P2-hm, pathogenicity island SaPI*sec* and phages Sa1*int* and Sa6*int* (Fig. 2).

All five MGEs were variably distributed across the CC22 phylogenetic tree and present in multiple clades, revealing an on-going process of horizontal acquisition and/or loss (Fig. 2). The relationship between the topology of the tree and MGE carriage was evaluated by measuring the phylogenetic signal (s2 value) of each element based on the phylogenetic generalized linear mixed model for binary data [25]. A value of s2 = 0 suggests no phylogenetic signal and so no association between the occurrence of a trait and phylogenetic structure (i.e. random distribution). On the contrary, increasing values of s2 denote a stronger phylogenetic signal and can be interpreted as a more stable vertical heritability of a trait. Among the five MGEs, the calculated s2 value ranged from 3 to 13. The lowest relative measure of phylogenetic signal was observed for P1-*ermC* (s2 = 3), followed by P2-hm (s2 = 5), Sa6*int* (s2 = 9), Sa1*int* (s2 = 11) and SaPI*sec* (s2 = 13). This implies that while the distribution of the analysed MGEs amongst CC22 isolates was correlated with their phylogenetic relationship, the strength of this association varied and was lower for the two plasmids P1-*ermC* and P2-hm in comparison with SaPI*sec*, Sa1*int* and Sa6*int*. This suggests that the distribution of these two plasmids was to a greater degree affected by independent horizontal acquisition events or sporadic losses than in the case of the chromosomally integrated MGEs, which are more frequently acquired vertically from a common ancestor.

**Fig. 3:**
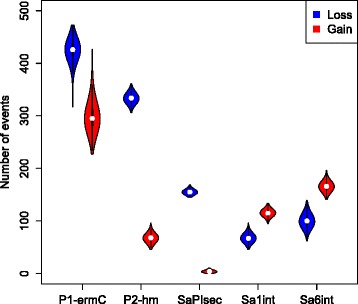
Number of MGE gain and loss events across the MRSA CC22 phylogeny. Ancestral state reconstruction for carriage of P1-*ermC*, P2-hm, SaPI*sec*, Sa1*int* and Sa6*int* was performed on MRSA CC22 phylogeny using stochastic character mapping. The violin plots show the distribution of estimated number of MGE gains and losses based on 1000 simulations

To further study the correlation between phylogeny and carriage of the five MGEs we conducted ancestral state reconstruction (ASR) of each element as a discrete trait (MGE presence or absence) by performing stochastic character mapping on the phylogenetic tree [26]. The estimated total number of transitions between the two states revealed that for P1-*ermC*, P2-hm and SaPI*sec* loss was more common, in contrast to Sa1*int* and Sa6*int*, where the gain event was more prevalent (Fig. 3). We investigated the estimated ancestral character states for all five MGEs at the internal node leading to the EMRSA-15 clade, which represents the vast majority of the analysed CC22 population. The node was predicted as positive for P1-*ermC* (mean = 0.935), P2-hm (mean = 0.993) and SaPI*sec* (mean = 1), but negative for Sa1*int* (mean = 0.001) and Sa6*int* (mean = 0.007). This is consistent with the estimated transition counts and shows that the heterogeneous MGE distribution across the CC22 phylogeny is driven by both sporadic losses (mostly P1-*ermC*, P2-hm and SaPI*sec*) as well as independent acquisition events (mostly Sa1*int* and Sa6*int*).

Plasmid P1-*ermC* demonstrated the highest number of overall state changes for both transition types, with the high number of estimated MGE losses accompanied by an equally frequent gain events. To rule out the possibility that the observed high rates of changes in the status of plasmid P1-*ermC* carriage was an artefact of sample processing leading to loss of this MGE during laboratory culture, we assessed the incidence of heterogeneous P1-*ermC* carriage amongst genetically identical isolates. We detected 22 pairs of isolates across the CC22 phylogeny with 0 SNPs difference at the core genome. Only a single pair revealed variable plasmid distribution (one of the isolates negative for P1-*ermC*), suggesting that in most cases plasmid carriage by highly related isolates is stable.

### Temporal variation in distribution of MRSA CC22 MGEs

**Fig. 4:**
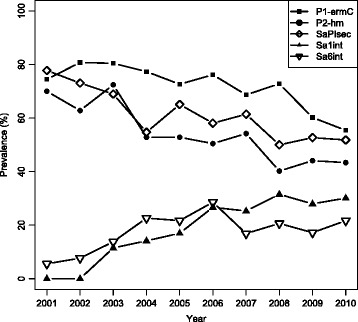
Prevalence of analysed MGEs amongst MRSA CC22 isolates between 2001 and 2010. MGEs that showed a significant temporal change in frequency were P1-*ermC*, P2-hm, SaPI*sec*, Sa1*int* and Sa6*int*. Prevalence shown as % of MRSA CC22 isolates in each year

We investigated the temporal dynamics of the five elements: P1-*ermC*, P2-hm, SaPI*sec*, Sa1*int* and Sa6*int*. All MGEs except for Sa1*int* were detected in every year between 2001 and 2010. In contrast, none of the isolates collected in either 2001 or 2002 carried phage Sa1*int*. Analysis of MGE frequency across years revealed a temporal trend, with a fall in prevalence of P1-*ermC*, P2-hm and SaPI*sec* but increase in frequency of Sa1*int* and Sa6*int*. The prevalence of P1-*ermC*, P2-hm and SaPI*sec* in CC22 between 2001 and 2010 fell from 74% to 55%, 70% to 43% and 78% to 52%, respectively (Fig. 4). In contrast, the carriage of Sa6*int* increased from 6% in 2001 to 22% in 2010. Isolates carrying Sa1*int* emerged in 2003 and increased in frequency from 11% (in 2003) to 31% in 2010. To test the significance of these shifts in MGE prevalence over time we used logistic regression to measure the effect and significance of the year of sampling on the presence of MGEs. For all elements, there was a significant association between the year of sampling and MGE carriage (*p* < 0.001 for P1-*ermC*, P2-hm, SaPI*sec* and Sa1*int*; *p* = 0.02 for Sa6*int*). We also tested whether MGE carriage status was correlated with lineage divergence as such relationship can indicate an evolutionary trajectory towards either MGE loss or gain. For this we measured the phylogenetic root-to-tip distance of all isolates and calculated the effect of this variable on MGE carriage status. A negative correlation between root-to-tip distance and presence of an element was observed for P1-*ermC*, P2-hm and SaPI*sec* (see Additional file 2: Figure S3), although this was more significant for P2-hm and SaPI*sec* (*p* < 0.001 and *p* = 0.004, respectively) than P1-*ermC* (*p* = 0.02). This is in agreement with our observation that P1-*ermC* can be frequently lost and re-acquired, which would likely undermine the strength of association between divergence and plasmid carriage status. In contrast a positive correlation between root-to-tip distance and MGE carriage was found for both Sa1*int* and Sa6*int* although it was significant only for the former (*p* < 0.001 and *p* = 0.21, respectively; see Additional file 2: Figure S3).

We attempted to investigate the mechanisms driving the observed temporal changes in the prevalence of MGEs in the CC22 population. Antimicrobial use is likely to constitute one of the main selective pressures driving the epidemiology of resistance-associated MGEs. Three elements showed a steady temporal decrease in prevalence including P1-*ermC*, which mediates resistance to erythromycin, a widely used drug in clinical practice. Over the ten-year period between 2001 and 2010 there was a steady drop in the use of erythromycin in the United Kingdom, a trend that has continued in recent years [27, 28]. However, this has been accompanied by an increase in the use of other macrolides, such as clarithromycin and azithromycin (see Additional file 2: Figure S4), demonstrating changes in prescription practices in accordance with clinical guidelines and improved tolerability. The *ermC* gene has been previously correlated with clarithromycin resistance in *S. epidermidis* and the use of this macrolide was found to select *ermC*-positive isolates [29]. It is therefore unclear whether changes in macrolide usage could have resulted in lower selective pressure on P1-*ermC* carriage leading to its temporal shedding. Nevertheless, the loss of P1-*ermC* over time correlated with a temporal reduction in the number of erythromycin resistant CC22 isolates (see Additional file 2: Figure S5). Out of the 678 CC22 isolates that were resistant to erythromycin, the majority carried the P1-*ermC* plasmid (653/678), confirming that this element represents the primary determinant of macrolide resistance in the analysed CC22 population. Although the mechanisms that have been driving the temporal loss of P1-*ermC* will require further scrutiny, more significantly this evolutionary change resulted in a lower prevalence of related phenotypic resistance.

**Fig. 5:**
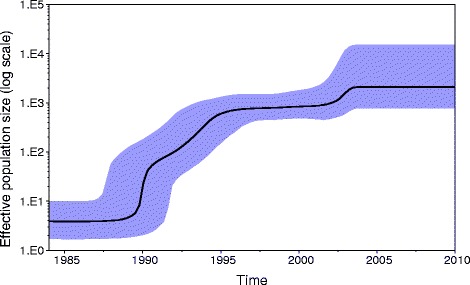
Bayesian skyline plot of inferred changes in the effective population size of MRSA CC22. The black line shows the median of the estimated effective population size whereas the background area represents the 95% highest posterior density intervals

Finally, we investigated whether the changes observed here in MGE composition had impacted on the genetic diversity of CC22 at the core genome. An analysis of pairwise SNP distances revealed a steady increase in CC22 heterogeneity between 2001 and 2010 (see Additional file 2: Figure S6). A Bayesian skyline plot of inferred historical changes in the effective population size of CC22 demonstrated that it rapidly expanded at the beginning of the 1990s, which coincides with the first reported isolation of the EMRSA-15 clone (Fig. 5). This growth continued and reached a plateau around the mid-1990s, with a further minor expansion between 2000 and 2005 followed by a plateau. This suggests that despite the apparent temporal loss of resistance- and virulence-associated MGEs, CC22 has maintained a stable population size over the corresponding time period.

## Discussion

Horizontal gene transfer represents one of the key mechanisms of *S. aureus* evolution, mediating acquisition of virulence and antimicrobial resistance-associated MGEs, which in turn drives the emergence of successful MRSA clones [3]. However, the MGE composition varies considerably between distinct MRSA populations and can also be heterogeneous amongst isolates of the same lineage [30, 31]. Here, we report novel insights into the evolution of the MGE content in MRSA population. Utilising WGS data we defined the content and prevalence of MGEs across the CC22 population, a globally disseminated and prevalent MRSA lineage. This revealed that the distribution of several variably occurring elements (P1-*ermC*, P2-hm, SaPI*sec*, Sa1*int* and Sa6*int*) was marked by a pattern of frequent carriage transitions involving multiple loss and gain events. We found a significant temporal pattern in the prevalence of these MGEs. This suggests the existence of selective pressures driving the long-term evolution of MGE carriage resulting in a measurable reduction or increase in the frequency of specific MGEs.

Elements that increased in prevalence were phage types Sa1*int* and Sa6*int*. Both have been reported to be infrequent in other *S. aureus* CCs [20, 32]. Little is known about the possible selective advantage of Sa1*int* or Sa6*int* acquisition. Carriage of virulence determinants has been reported previously for Sa1*int,* namely the exfoliative toxin gene *eta* [20]*.* However, this gene was not found in any of the analysed CC22 isolates. In contrast, MGEs that showed a steady temporal decline in prevalence across the CC22 population between 2001 and 2010, namely plasmids P1-*ermC* and P2-hm as well as pathogenicity island SaPI*sec*, were all vectors of either antimicrobial resistance or virulence genes. Plasmid P1-*ermC* encodes the erythromycin ribosomal methylase ErmC, conferring resistance to erythromycin and additionally to clindamycin if the *ermC* gene is constitutively expressed due to mutations in its regulatory region [33]. The *ermC* gene is a common determinant of erythromycin resistance in *S. aureus* and other staphylococci, and is mainly found on small multi-copy plasmids such as P1-*ermC* [34]. The successful dissemination of this plasmid type is evident in its wider distribution among other MRSA lineages analysed here. This may reflect the high levels of macrolide use in the UK, the third most common group of antibiotics prescribed in clinical practice [35]. Consistently, the absence of this element in certain *S. aureus* populations, such as isolates from companion animals, has been linked with lack of selective pressure due to lower erythromycin usage [36]. In contrast to P1-*ermC*, the second plasmid P2-hm was not observed outside the CC22 lineage, suggesting that the element may be CC22-specific. This plasmid carried a cluster of heavy metal resistance genes consisting of *cadA*, *copB*, *mco* and *arsAB*. Interestingly, an integrated plasmid carrying the same cluster of heavy metal resistance genes has been also found in EMRSA-16 [22], indicating an association of these resistance genes with distinct HA-MRSA lineages. Occurrence of heavy metal resistance genes in bacterial pathogens has been linked with metal contamination in the environment as well as co-selection due to antibiotic co-resistance or cross-resistance [37]. Carriage of genes expressing copper detoxification mechanisms such as CopB ATPase transporter and multi-copper oxidase (MCO) may be also associated with adaptation towards enhanced survival of bacterial pathogens within the host. It has been observed that copper might play an important role in innate immunity by enhancing bactericidal activity of macrophages, and resistance to copper has been associated with virulence in various bacterial pathogens such as *Mycobacterium tuberculosis*, *Streptococcus pneumoniae* and *Neisseria gonorrhoeae* [38–41]. The *S. aureus* chromosome commonly contains a conserved copper resistance operon composed of *copA* and *copZ* genes [42]. Carriage of plasmid-associated *copB* and *mco* genes further enhances copper tolerance in *S. aureus*, resulting in a hyper-copper-resistance phenotype that might aid survival in copper-rich environments [43]. Similar to P2-hm, SaPI*sec* was restricted to CC22 isolates. The enterotoxin C (SEC), encoded by the *sec* gene, belongs to the family of staphylococcal pyrogenic exotoxins that act as superantigens, inducing a non-specific T cell activation [44]. Staphylococcal enterotoxins such as SEC also represent gastrointestinal toxins and have mostly been described as causative factors of staphylococcal food poisoning [45]. Recently, SEC has also been shown to be a critical virulence factor in a rabbit model of staphylococcal infective endocarditis and sepsis, both through super-antigenicity and direct interaction with endothelial cells [46]. Another study demonstrated that vaccination against SEC provides protection against *S. aureus* induced necrotizing pneumonia in a rabbit model of the disease, further suggesting that SEC plays an important role in *S. aureus* invasive disease [47].

Together, the three MGEs (P1-*ermC*, P2-hm and SaPI*sec*) represent elements that are likely to provide an initial selective advantage by altering the pathogenicity and survival of invasive *S. aureus*. It is striking to observe a common temporal trend of diminishing prevalence for all three elements in CC22. Also, a temporal reduction in the prevalence of an antibiotic resistance element such as P1-*ermC* represents a unique evolutionary trajectory for a hospital-associated pathogen. Furthermore, the loss of each element occurred across the CC22 phylogeny with multiple presence-to-absence transitions. This indicates that the evolutionary trend towards falling frequency of MGEs involved the broader CC22 population rather than being driven by expansion of MGE-negative isolates. It has been suggested previously that the success of MRSA CC22 might be partly driven by its ability to rapidly gain and lose MGEs under changing environmental conditions [31]. Furthermore, the temporal reduction of the MGE content appears to be specific to CC22. A broader analysis of MGEs amongst non-CC22 lineages was not conducted due to small sample sizes for other CCs, but we observed that the vast majority of CC30 isolates carried the previously mentioned EMRSA-16-associated heavy metal resistance plasmid at an overall prevalence of 97%. Similarly, the CC30-associated pathogenicity islands, SaPI-2 and SaPI-4, were detected in at least 96% of CC30 isolates, demonstrating stable carriage by the study collection.

It has been observed previously that the epidemiology of major MRSA lineages resembles a wave of expansion followed by population equilibrium and then decline [48]. While MRSA CC22 remains the dominant lineage of HA-MRSA in the UK, the epidemiology of MRSA in the UK healthcare setting has been shifting. A fall in prevalence of EMRSA-16 has been widely reported together with a decline in MRSA isolation rates [13, 19, 49, 50]. This has been linked with an implementation of various infection control measures across the UK hospitals [51, 52]. However, others reported that the change in MRSA epidemiology began prior to the onset of these infection control initiatives and was clone-specific [13, 50]. Changing biology of the MRSA population might have played a more critical role in the declining rates of hospital MRSA infections [50]. The changing MGE composition of CC22 should therefore be considered in the context of the shifting MRSA epidemiology. It is unclear whether the loss of distinct MGEs reflects a positively-selected evolutionary adaptation or loss of non-essential elements due to diminishing selective pressure. The latter might constitute changes in antimicrobial prescription practices. An association between a reduced consumption of macrolides and an increase in frequency of susceptible isolates in MRSA population in a hospital setting has been observed [52]. Other factors may be at play, such as the decline in prevalence of a competitive clone, namely EMRSA-16, providing that the analysed here MGEs have played an important role in out-competing other prevalent MRSA lineages. A lower selective pressure to maintain specific virulence and antimicrobial resistance associated MGEs might also relate to the wider changes in MRSA epidemiology. Our data shows that MRSA CC22 has maintained a stable effective population size between 2001 and 2010 despite dropping prevalence of hospital acquired MRSA infections suggesting that this lineage is likely well adapted to survival outside of clinical setting. There is some evidence that the EMRSA-15 clone might be common in the MRSA population outside of hospital environment in the UK, based on isolates from community associated-MRSA infections [53, 54]. An asymptomatic carriage of this clone amongst healthy young individuals with no risk association was also reported in Ireland [55]. The changing composition of MGE might therefore reflect loss of elements that are less critical for successful carriage or transmission within a community.

## Conclusions

The CC22 MRSA lineage has a flexible MGE composition based on the heterogeneous distribution of several MGEs across its phylogeny, which is associated with high frequency of MGE loss and acquisition events. The evolutionary pattern of MGE flux correlated with the temporal prevalence of studied MGEs. The evolutionary changes observed here further indicate that CC22 can rapidly alter its MGE composition, which in turn may contribute to the successful dissemination and persistence of this clone. In addition, the observed drop in prevalence of an antimicrobial resistance-associated MGE over time reveals a novel evolutionary trajectory for an important hospital-associated bacterial pathogen, which in turn might demonstrate adaptation to survival outside of hospital setting.

## Methods

### Bacterial isolates

The 1193 *S. aureus* bacteraemia isolates studied here derived from two sources. A total of 1013 isolates were from a collection described previously that spanned the period from 2001 to 2010 [56]. In brief, these were predominantly MRSA (*N* = 990), collected by the British Society for Antimicrobial Chemotherapy (BSAC) who coordinate antimicrobial resistance surveillance across the United Kingdom and Ireland [57]. Diagnostic microbiology laboratories submit a defined number of consecutive but non-duplicate *S. aureus* isolated from blood cultures each year. A total of 47 centres contributed *S. aureus* bacteraemia isolates between 2001 and 2010. A further 180 non-duplicated isolates (all but one MRSA) associated with bloodstream infection were collected from hospitals in the East of England over the same time period (apart from during 2008 or 2009).

### Whole-genome sequencing, assembly and annotation

Genomic DNA was isolated using the Qiagen QIAxtractor system. Tagged DNA libraries were created using a method adapted from a standard Illumina Indexing protocol, as described previously [16]. Whole-genome sequencing was performed on the Illumina HiSeq 2000 platform with 100 bp paired-end reads. Annotated assemblies were produced as previously described [58]. Briefly, de novo assembly of whole genome sequences was performed using Velvet v1.2 [59] with Velvet Optimiser v2.2.5 [60]. Contigs were scaffolded with SSPACE [61] and sequence gaps closed using GapFiller [62]. The assembled contigs were annotated using Prokka v1.11 [63] and *S. aureus* specific database from RefSeq [64].

### Genotyping and phylogenetics

Multi-locus sequence typing based on the previously described *S. aureus* typing scheme [6] was performed on assembled genomes using an in-house pipeline, MLST check [65]. To query evolutionary relationships between isolates, single nucleotide polymorphisms (SNPs) were detected by mapping paired-end reads against the *S. aureus* HO 5096 0412 reference genome [9], using SMALT version 0.7.4 [66]. After excluding MGEs from the generated alignment, an approximately-maximum-likelihood phylogenetic tree was generated using FastTree [67]. Clonal complex specific phylogenies were further reconstructed for CC22 and CC30. The alignment based on HO 5096 0412 genome was used for CC22, whereas isolates belonging to CC30 were re-mapped against the MRSA252 reference genome [22]. For each CC a core genome alignment was created by excluding MGE regions, variable sites associated with recombination (detected with Gubbins [68]) and sites with more than 5% proportion of gaps (i.e. sites with an ambiguous character). Maximum likelihood (ML) phylogenetic trees were generated using RAxML version 7.8.6 [69] based on the generalised time reversible (GTR) model with GAMMA method of correction for among site rate variation and 100 bootstrap replications [8]. Phylogenetic trees were annotated using Evolview [70, 71]. CC22 population demographic history was estimated based on a subset of 100 isolates using Bayesian Evolutionary Analysis Sampling Trees (BEAST) software package v1.8.2 [72]. The MCMC chain was run for 50 million generations, sampling every 1000 states using the HKY four discrete gamma substitution model. The Bayesian population size model was tested with strict and lognormal-relaxed molecular clocks, each run in triplicate. Only the strict clock model completed with good convergence of chains and a suitable effective sample size parameter. The Bayesian skyline plot was generated with Tracer v1.5.

### MGE analysis

Genome fragments representing MGEs were defined as described previously [8]. Briefly, the presence of variably distributed sequence fragments of >1 kbp across assemblies of all CC22 isolates was determined, followed by annotation and Basic Local Alignment Search Tool (BLAST) analysis to determine the class of putative MGE. Contigs were considered to represent a putative plasmid if presence of a *rep* gene was detected or if the element shared sequence similarity with a previously identified plasmid, as defined by a BLAST search. Sequence fragments that were >1 kbp but contained a single CDS only were excluded from further analyses as the type of MGE could not be defined. A mobile genetic element database was collated from the defined sequence fragments and applied to screen the distribution of each sequence across all isolates using the short-read sequence typing tool (SRST2) [73]. If the presence of an element was detected based on short read mapping, this was further verified by assembly alignment with the use of MUMmer [74]. MGEs were recorded as present whether found assembled on a single contig or occurring as fragmented assemblies.

### Statistical analysis

Analysis of trait evolution pattern for the distribution of MGEs was conducted on the ML phylogeny of CC22. Root-to-tip distances were calculated using the TempEst tool [75]. The phylogenetic signal of MGE distribution as well as the logistic regression of MGE distribution as a categorical binary value (presence/absence) and predictor variables (year of sampling, root-to-tip divergence) was measured using *binaryPGLMM* function from R package *ape*. The ancestral state reconstruction (ASR) was performed with stochastic character mapping using the *make.simmap* function of the R package *phytools*. Two evolutionary models were compared: equal rates (ER) and all-rates-different (ARD) by estimating the Akaike information criterion (AIC) using the *fitDiscrete* function of R package *geiger*. The ARD model gave a better fit and was applied in ASR. The reconstructions were run with 1000 simulations.

## Additional files

Additional file 1: Table S1. Summary of isolates analysed in this study. (XLSX 99 kb)

Additional file 2: Figure S1. Prevalence of isolates representing CC5, CC22 and CC30 between 2001 and 2010. Shown as % of *S. aureus* isolates in each year. Error bars show 95% confidence intervals. **Figure S2** (A, B). Unrooted maximum likelihood tree of *S. aureus* isolates belonging to CC22 (A) or CC30 (B). Both lineages were composed predominantly of EMRSA isolates; red branches show clades representing the EMRSA-15 (A) and EMRSA-16 (B) clones. **Figure S3.** Relationship between the probability of MGE carriage and the root-to-tip distance in MRSA CC22 isolates. Curve based on prediction from logistic regression model. **Figure S4.** Annual macrolide prescription items in England between 2001 and 2010. A prescription item refers to a single item prescribed on a prescription form. Copyright NHSBSA 2013. This information is licenced under the terms of the Open Government Licence: http://www.nationalarchives.gov.uk/doc/open-government-licence/version/3. **Figure S5.** Prevalence of P1-*ermC* carriage and erythromycin resistance in MRSA CC22 isolates between 2001 and 2010. Shown as % of MRSA CC22 isolates in each year. **Figure S6.** Median of pairwise SNP distances between MRSA CC22 isolates each year between 2001 and 2010. (DOCX 1420 kb)

Additional file 3: Table S2. Summary of MGEs identified in 923 MRSA CC22 isolates, describing distribution across the CC22 and non-CC22 isolates. (XLSX 39 kb)

## Abbreviations

ACME: Arginine catabolic mobile elements
CC: Clonal complex
MGE: Mobile genetic element
ML: Maximum likelihood
MLST: Multi-locus sequence typing
MRSA: Methicillin-resistant *Staphylococcus aureus*
SCC*mec*: Staphylococcal cassette chromosome *mec*.
