# Supplementary material for: Evolution of mobile genetic element composition in an epidemic methicillin-resistant Staphylococcus aureus: temporal changes correlated with frequent loss and gain events
Source: BMC Genomics. 2017 Sep 4;18:684. doi: 10.1186/s12864-017-4065-z (PMC5584012; doi:10.1186/s12864-017-4065-z)
Supplement: Supplementary file 2 — Prevalence of isolates representing CC5, CC22 and CC30 between 2001 and 2010. Shown as % of S. aureus isolates in each year. Error bars show 95% confidence intervals. Figure S2 (A, B). Unrooted maximum likelihood tree of S. aureus isolates belonging to CC22 (A) or CC30 (B). Both lineages were composed predominantly of EMRSA isolates; red branches show clades representing the EMRSA-15 (A) and EMRSA-16 (B) clones. Figure S3. Relationship between the probability of MGE carriage and the root-to-tip distance in MRSA CC22 isolates. Curve based on prediction from logistic regression model. Figure S4. Annual macrolide prescription items in England between 2001 and 2010. A prescription item refers to a single item prescribed on a prescription form. Copyright NHSBSA 2013. This information is licenced under the terms of the Open Government Licence: http://www.nationalarchives.gov.uk/doc/open-government-licence/version/3. Figure S5. Prevalence of P1-ermC carriage and erythromycin resistance in MRSA CC22 isolates between 2001 and 2010. Shown as % of MRSA CC22 isolates in each year. Figure S6. Median of pairwise SNP distances between MRSA CC22 isolates each year between 2001 and 2010. (DOCX 1420 kb) [file 12864_2017_4065_MOESM2_ESM.docx]

**Figure S1.** Prevalence of isolates representing CC5, CC22 and CC30 between 2001 and 2010. Shown as % of *S. aureus* isolates in each year. Error bars show 95% confidence intervals.

**Figure S2 (A, B).** Unrooted maximum likelihood tree of *S. aureus* isolates belonging to CC22 (A) or CC30 (B). Both lineages were composed predominantly of EMRSA isolates; red branches show clades representing the EMRSA-15 (A) and EMRSA-16 (B) clones.

**Figure S3.** Relationship between the probability of MGE carriage and the root-to-tip distance in MRSA CC22 isolates. Curve based on prediction from logistic regression model.

**Figure S4.** Annual macrolide prescription items in England between 2001 and 2010. A prescription item refers to a single item prescribed on a prescription form. Copyright NHSBSA 2013. This information is licenced under the terms of the Open Government Licence: http://www.nationalarchives.gov.uk/doc/opengovernment-licence/version/3

**Figure S5.** Prevalence of P1-*ermC* carriage and erythromycin resistance in MRSA CC22 isolates between 2001 and 2010. Shown as % of MRSA CC22 isolates in each year.

**Figure S6.** Median of pairwise SNP distances between MRSA CC22 isolates each year between 2001 and 2010.
